# Evaluating health research priority-setting in low-income countries: a case study of health research priority-setting in Zambia

**DOI:** 10.1186/s12961-018-0384-z

**Published:** 2018-11-07

**Authors:** Lydia Kapiriri, Corinne Schuster-Wallace, Pascalina Chanda-Kapata

**Affiliations:** 1 Department of Health, Aging and society, McMaster University, 1280 Main Street West, Hamilton, ON Canada; 2 McMaster University, 30 Huntingwood Avenue, Dundas, ON Canada; 3 Department of Diseases Surveillance Control and Research, Ministry of Health, Lusaka, Zambia

**Keywords:** Health research priority-setting, Stakeholder engagement, Evaluation, Low-income countries

## Abstract

**Abstract:**

Priority-setting (PS) for health research presents an opportunity for the relevant stakeholders to identify and create a list of priorities that reflects the country’s knowledge needs. Zambia has conducted several health research prioritisation exercises that have never been evaluated. Evaluation would facilitate gleaning of lessons of good practices that can be shared as well as the identification of areas of improvement. This paper describes and evaluates health research PS in Zambia from the perspectives of key stakeholders using an internationally validated evaluation framework.

**Methods:**

This was a qualitative study based on 28 in-depth interviews with stakeholders who had participated in the PS exercises. An interview guide was employed. Data were analysed using NVIVO 10. Emerging themes were, in turn, compared to the framework parameters.

**Results:**

Respondents reported that, while the Zambian political, economic, social and cultural context was conducive, there was a lack of co-ordination of funding sources, partners and research priorities. Although participatory, the process lacked community involvement, dissemination strategies and appeals mechanisms. Limited funding hampered implementation, monitoring and evaluation. Research was largely driven by the research funders.

**Conclusions:**

Although there is apparent commitment to health research in Zambia, health research PS is limited by lack of funding, and consistently used explicit and fair processes. The designated national research organisation and the availability of tools that have been validated and pilot tested within Zambia provide an opportunity for focused capacity strengthening for systematic prioritisation, monitoring and evaluation. The utility of the evaluation framework in Zambia could indicate potential usefulness in similar low-income countries.

## Background

Several international organisations have responded to the call to bridge the existing inequities in health research funding whereby less than 10% of global funding for research is spent on diseases that afflict more than 90% of the world’s population (i.e. the 10/90 gap) by increasing their funding for global health research [1]. Several international organisations, for example, the Canadian government, through Global Affairs Canada, the International Development Research Council and the Canadian Institute for Health Research, are committed to strengthening health systems in low-income countries and supporting research infrastructure [2]. Unfortunately, very few countries have met the committed research funding targets [1]. It is essential that these resources are allocated to solve the most pressing global health problems. This allocation is even more critical given the push to base policy decisions on the best-available scientific evidence. It is reasonable to assume that interventions for which evidence is available have a higher chance of being considered in priority-setting (PS), and thus being implemented. Research priorities that address the leading causes of disease burden would contribute to reducing the burden and improving population health [3].

PS for health research has emerged as an important field in response to the gap between the health research funding and the research needs, as well as the disconnect between the health policy information needs and the funded research [4, 5]. At the national level, PS is the first, and often missing, step in connecting research with policy and practice needs, hence reducing research waste (by ensuring that the research that is conducted is well aligned with the policy and practice needs). It presents an invaluable opportunity for the relevant stakeholders to convene, based on the best-available evidence and criteria, to identify and create a ranked list of priorities that captures the country’s knowledge needs [3, 6, 7]. To facilitate oversight for PS for health research, several international organisations have supported the establishment of National Health Research Organisations [8–11]. Furthermore, there is an increasing body of literature on the tools that can be used to guide PS [3–12]. Approaches such as the Child Health and Nutrition Research Initiative method, the James Lind Alliance method, the Combined Approach Matrix method and the Essential National Health Research method have been developed and used to guide health research prioritisation in different contexts [13]. However, the literature on studies evaluating PS processes in low- and middle-income countries is limited.

Health research is an integral part of the Zambian health system. Zambia established a National Health Research Advisory Committee in 2005 to provide ad hoc support to the Zambian Ministry of Health on all matters relating to health research [13]. Following this, in 2008, the government approved the National Health Research Policy, with one of the policy measures including the formation of a national body to coordinate health research. This body, the National Health Research Authority of Zambia, established by an Act of Parliament in 2013, was established with the responsibilities of coordinating health research in Zambia, overseeing research ethics, conducting PS exercises, facilitating capacity strengthening for all stakeholders involved in health research, advocating for the role of research in the policy process, and funding some primary research [14–18].

Concurrent with the setting up of the health research infrastructure, Zambia has undertaken up to six health research PS exercises that were led by various organisations and stakeholders. The first exercise was conducted by the National Health Research Advisory Committee (1998 to 1999); subsequently, health research priorities were identified as part of the National Health Strategic Plan (2006–2011). Another initiative was led by the National Science and Technology Council, and yet another exercise was facilitated by the Zambia Forum for Health Research. Furthermore, the Ministry of Health, in partnership with WHO, identified research priorities for maternal, neonatal and child health. Most recently (in 2018), the National Health Research Authority facilitated a national prioritisation process in which efforts were made to ensure that the research priorities were in line with the key national policy documents, namely the Seventh National Development Plan 2017–2021 and the Ministry of Health Strategic Plan 2017–2021. This process resulted in a long list of priorities being generated (summarised in Table 1) [16].

**Table 1:** Summary of the most recent Zambia research priorities [16]

Category according to the Essential Care Package	Number of research priorities identified
Reproductive, maternal, neonatal, child, adolescent health and nutrition	Reproductive (9), maternal (8), neonatal (6), child (9), adolescent health (11) and nutrition (17)
Communicable diseases	HIV (60), tuberculosis (17), hepatitis B (5), neglected tropical diseases (3), malaria (66)
Non-communicable diseases	General priorities (8), cancer (13), mental health (3), hormonal- diabetes (1), road safety (2), surgery (2).
Disease outbreaks and epidemics control	4
Biomedical sciences	3
Health promotion, social determinants, environmental health, primary healthcare, community health	11
Health systems	Human resources (7), essential drugs and diagnostics (9) infrastructure (1), information (2), financing (3), leadership (3), service delivery (1), innovation (5), traditional, complimentary, alternative and alternative medicine (5)

While a number of research priority initiatives have been implemented within the Zambian health sector within this period, there has been no systematic process to evaluate the degree to which PS for these initiatives was successful [15, 16]. Furthermore, addressing shortcomings in the health research PS processes contributes to ensuring that the relevant research is conducted and that decision-makers in low-income countries have relevant context-specific evidence to guide their decision-making. This paper fills this gap by describing and evaluating health research PS in Zambia using a validated evaluation framework.

The objectives of this paper are the following:

1. To describe and evaluate previous cases of PS for health research in Zambia from the perspectives of key stakeholders using an internationally validated evaluation framework.
2. To identify lessons of good practice to be shared with other low-income countries as well as areas for improvement.
3. To reflect on the applicability of the validated framework for evaluating PS for health research in Zambia.

## Methods

### The evaluation framework

While being primarily developed for evaluating PS for health interventions, Kapiriri and Martin (2010)’s framework was modified and validated for evaluating PS for health research [forthcoming]. The 2010 framework was developed based on the current literature on good practices in PS, as well as a Delphi process with stakeholders involved in health research PS and funding. The framework identifies parameters that are relevant in evaluating PS. These relate to the PS context, the pre-requisites, the PS process, implementation of the priorities, outcome and impact (Table 2). These are grouped under internal and external parameters relating to the PS institution, as well as immediate and delayed parameters indicating the timeframe for evaluating the parameters. For each parameter, objectively verifiable indicators and means of verification are specified, e.g. objectively verifiable indicators for the parameter ‘contributing to meeting the Ministry of Health goals’ include disability-adjusted life years or mortality rates, and health management information reports can be used as the means of verification. This framework provides adequate detail to enable policy-makers to identify critical barriers and facilitators of effective PS [19].

**Table 2:** Framework for evaluating priority-setting

Domains	Parameters of successful priority-setting	Objectively verifiable indicators	Means of verification
Contextual factors	Conducive political, economic, social and cultural context	Relevant contextual factors that may impact priority-setting	Follow-up intermittent interviews with local stakeholders, systematic longitudinal observations, relevant reports, Media
Pre-requisites	Political will	Degree to which the politicians support the set priorities	Follow-up intermittent interviews with local stakeholders, systematic longitudinal observations, relevant reports, Media
	Resources	Budgetary and human resource allocation to the health research	National budget documents
	Legitimate and credible institutions	Degree to which the priority-setting institutions can set priorities, public confidence in the institution	Stakeholder and public interviews
	Incentives	Material and financial incentives	National budget documents
The priority-setting process	Stakeholder participation	Number of stakeholders participating, number of opportunities, each stakeholder gets to express opinion	Observations/minutes at meetings, media reports, special reports
	Use of clear priority-setting process/tool/methods	Documented priority-setting process and/or use of priority-setting framework	Observation/minutes at meetings, media reports, special reports
	Use of explicit relevant priority-setting criteria	Documented/articulated criteria	Observations/minutes at meetings, media reports, special reports
	Use of evidence	Number of times available data is resourced/number of studies commissioned/existing strategies to collect relevant data	Observations/minutes at meetings, media reports, special reports
	Reflection of public values	Number and type of members from the general public represented, how they are selected, number of times they get to express their opinion, proportion of decisions reflecting public values, documented strategy to enlist public values, number of studies commissioned to elicit public values	Observations/minutes at meetings, study reports, meeting minutes and strategic plans
	Publicity of priorities and criteria	Number of times decisions and rationales appear in public documents	Media reports
	Functional mechanisms for appealing the decision	Number of decisions appealed, number of decisions revised	Observations/minutes at meetings, media reports, special reports
	Functional mechanisms for enforcement	Number of cases of failure to adhere to priority-setting process reported	Observations/minutes at meetings, media reports, special reports
	Efficiency of the priority-setting process	Proportion of meeting time spent on priority-setting, number of decisions made on time	Observations/minutes at meetings, annual budget documents, health system reports
Implementation of the set priorities	Decreased dissentions	Number of complaints from stakeholder	Meeting minutes, media reports
	Allocation of research resources according to priorities	Degree of alignment of resource allocation and agreed-upon priorities, times budget is re-allocated from less prioritised to highly prioritised areas, stakeholder satisfaction with the decisions	Annual budget reports, evaluation documents
	Decreased resource wastage/misallocation	Proportion of budget unused or allocated to non- priority research	Budget documents, research and evaluation reports
	Improved internal accountability/reduced corruption	Number of publicised resource allocation decisions	Evaluation reports, stakeholder interviews, media reports
	Increased stakeholder understanding, satisfaction and compliance with the priority-setting process	Number of stakeholders attending meetings, number of complaints from stakeholder, percentage of stakeholder that can articulate the concepts used in research priority-setting and appreciate the need for priority-setting	Observations/minutes at meetings, special reports, stakeholder satisfaction survey, media reports, stakeholder interviews, evaluation reports
	Improved internal accountability/reduced corruption	Number of publicised resource allocation decisions	Evaluation reports, stakeholder interviews, media reports
	Strengthening of the PS institution	Indicators relating to increased efficiency, use of data, quality of decisions and appropriate resource allocation, percentage of stakeholders with the capacity to set priorities	Training reports, evaluation reports, budget documents
	Impact on research institution goals and objectives	Percentage of of research institution objectives met that are attributed to the priority-setting process	Evaluation reports, special studies
Outcome and impact	Impact on health policy and practice	Changes in health policy to reflect identified priorities	Policy documents
	Achievement of health system goals	Research contribution to achievement of health system goals	Ministry of Health documents, Demographic and Health Surveys, commissioned studies
	Improved financial and political accountability	Number of publicised financial resource allocation decisions, number of corruption instances reported, percentage of the public reporting satisfaction with the process	Reports, media reports, interviews with stakeholders
	Increased investment in the health sector and strengthening of the healthcare system	Proportion increase in the health research budget, percentage of the public/researchers reporting satisfaction with the health research system	National budget allocation documents

Prior to its use in this study, the framework was validated by stakeholders engaged in health research PS at the global level and within low-income countries. During the validation process, respondents were asked to indicate their perception of the degree of importance of the parameters in the framework when evaluating health research prioritisation. Most of the respondents validated almost all the parameters as very important when evaluating health research prioritisation with the exception of three parameters (availability of incentives, increased efficiency and availability of appeals mechanisms) [Kapiriri, forthcoming].

#### Study design

This was a qualitative study involving interviews with stakeholders at the national and provincial levels who had been involved in or were identified as knowledgeable of health research PS in Zambia.

##### Sampling

The respondents were purposefully sampled by virtue of having been involved in at least one of the prioritisation processes. The participants’ list from the most recent prioritisation process provided the initial list of contacts. Once they had been interviewed, they provided contacts for additional potential respondents who were also interviewed. We stopped identifying respondents once the respondents began to consistently identify respondents we had already interviewed. While we conducted a total of 35 interviews, 28 were complete and hence included in this analysis.

##### Data collection

Key informant interviews were conducted between 2015 and 2016. An interview guide, based on the evaluation framework, was used to collect the data. However, this was used with flexibility in order to capture any new themes. Face-to-face interviews were conducted by a trained research assistant. The interviews lasted an average of 40 min and were audio-recorded by permission from the respondents. The recorded interviews were transcribed verbatim.

##### Analysis

A modified thematic approach was used. Transcripts were read through manually, and broad emerging themes identified. Following this, themes were compared to the evaluation framework and additional codes were identified based on the parameters in the framework. The combination of the initial themes and the codes identified from the framework were used as a basis for re-coding the interviews in NVIVO 10. Once coded, the emerging themes were mapped onto the evaluation framework to evaluate PS for health research in Zambia and to assess the degree to which they were consistent with the framework. Any variations were noted. Further analysis involved identifying lessons that are transferrable and could be shared with similar low-income countries.

The analysis related to the framework involved assessing the degree to which the study was able to gather the required information using the means of verification as proposed in the evaluation framework. The parameters that could not be assessed were identified and explanations for this were discussed.

## Results

The results are based on analysis of 28 in-depth interviews (consisting of 4 donors, 10 government/Ministry of Health officers, and 1 hospital, 2 non-governmental organisation (NGO) and 11 university representatives). While we endeavoured to interview respondents from sub-national levels, the majority of the respondents worked at the national level (either with the Ministry of Health, research funding organisations and researchers from the university). These had directly participated in or were knowledgeable of the previous health research prioritisation efforts.

The results were organised according to the domains and parameters in the evaluation framework. Where necessary, additional sub-headings were used to denote those themes that were not reflected in the framework. The themes, in accordance to the domains in the framework, include Contextual factors, Pre-requisites, PS process, Implementation, and Outcome and impact.

### Contextual factors

According to the validated evaluation framework, PS is influenced by the context within which it occurs. A conducive political, economic, social and cultural environment would support successful prioritisation. These factors were reflected on in the interviews, as follows:

“*The policy, legal and the funding framework. Those definitely are the key* [factors] *that will affect* [PS]*, because you can set these research priorities and then the policy is in a different direction; or the funding is not there to implement whatever the priorities you have set*.” [#19]

However, while respondents reported that the key political, economic, social and cultural ingredients for a conducive environment exist, six respondents across government, university and NGO sectors noted a lack of co-ordination, for example, of funding sources, partners and research priorities themselves.

“*Because, you see, you have a strategic plan at Ministry and at the university. And if you don’t link us to your priority-setting, we are not going to do it. So, we need to see how we are going to link the priorities set to institutional priority-setting and also other non-organisation, because they do a lot of research. So, a mechanism should be developed to link the priority-setting for Ministry to other organisations*.” [#16]

“*I do think that there’s a lot of fragmentation, and I think that perhaps, while resources are always a challenge; that sometimes is because there’s almost a scattergun approach, with lots of different active groups, wanting to do different things. And they’re all pulling in different directions. Sometimes it would be helpful to have more ownership from government, saying ‘This is the area that is our top priority’ to help focus those activities.*” [#2]

There was a perception, by the respondents, that the political context may not be conducive due to political interests and fear:

“*It’s a huge risk because, as you know, a lot of the things we do on the academic side always have big caveats – a lot of assumptions – built into it. And so policy-makers are sometimes scared. Say, look, this is just a number on paper. If I make the decision and allocate public money on the basis of this, there could be political fall-out*.” [#26]

### Pre-requisites

The framework identifies three pre-requisites for successful PS, namely political will, availability of human and financial resources and incentives, and a PS institution with the capacity to set priorities.

#### Political will

Nineteen respondents representing government and universities agreed that political will and the role of government are core elements of the health research PS framework and that there is a need for and some evidence of political support for health research in Zambia.

“*… If we have got a government that emphasises the importance of research, evidence, etc., in the next 20 years, we’ll be talking about something to do with… something related to development.*” [#14]

However, several respondents expressed frustration that, in spite of the political support, the lack of clear leadership (that would guide the research activities and/or deliver impact) has resulted in fragmentation and silo approaches within and between ministries. They also reported a lack of clear delineation of roles and responsibilities, despite the existence of several key Government policies and plans including the Health Research Act, the Science and Technology Act, the 6th National development Plan, and the National Science and Technology Council.

“*I think there’s a lot of political will out there it’s just we need to translate that into actual resources*.” [#25]

#### Human resources

While all except four respondents recognised the need for human resources with the capacity to access grants and conduct research, there were conflicting responses with regards to the existence of these resources. Many (*n* = 16) respondents, predominantly in the donor and university sectors, believed the capacity existed, or was improving, as evidenced by some researchers receiving international grants and recognition.

“*We’re extremely good at writing proposals. We have capacity to conduct research*.” [#15]

However, contrary to the above respondents, other respondents, predominantly from NGO and government sectors, identified some challenges related to human resources. For example, the Zambian researchers are not always successful in accessing competitive funding. This was in part attributed to the lack of access to research infrastructure, such as fully equipped laboratories and an inability to retain staff. Another human resources gap identified was the capacity to communicate (publish papers), utilise and implement the research findings. Furthermore, many respondents were concerned with staff turnover, lack of government continuity (particularly at the local and regional levels) and policy changes.

“*Management change, follow-up of implementation, and institutional memory. These three, if you can address them, you’re going to improve the dream, the vision for research. Nobody picks it up*.” [#17]

#### Financial resources

The lack of in-country resources allocated to health research was identified by all respondents as a critical challenge to health research and domestic PS. While it was acknowledged that the government has started to allocate money to research in the last 5 years, it is not sufficient and has not achieved the pan-African-agreed target of 1% of GDP. This sets up conditions for competition that can undermine collaboration between researchers. It is also seen to be frustrating when research funds become available, only to have been pre-allocated, or to be clawed back for competing higher priority needs such as emergency investments in the health system.

The lack of research funding was seen to threaten not only research-informed health system interventions and improvements, but also domestic research capacity, particularly for graduate students, who were seen as the next generation of health researchers in the country. Furthermore, funds for health research were distributed over multiple ministries, including the Ministries of Health and Science and Technology, creating inefficiencies for the government to manage funds and track outputs and impact, and making it difficult for researchers to know where to apply to access the funds or where to best share research findings.

“*Priority-setting is a process that has to be thought through and has to be detailed and resources have to be put into these priorities in order to achieve that. If you come up with a real good plan and then you can’t implement it because what needs to go into it is not put in place, there is no way you are going to achieve it. So this is where we are at…. Did we have the people who had the capacity to come up* [with] *a beautiful tool? Yes. Was there money to actually implement that beautiful tool? No*.” [#18]

#### Legitimate and credible institutions with the necessary capacity

Respondents were divided with regards to the availability of a PS institution with capacity to identify research priorities. Six respondents, from the Ministry of Health and the university, reported that Zambia is home to legitimate and credible institutions and individuals, including the national research body articulated in the 2013 Act, even though the national research body was so in name only (with the Directorate of public health undertaking its functions) until 2015. The PS process was even seen to build capacity in and of itself, although not everyone is aware of PS processes. The Ministry of Health, in particular, was seen to “*have more legitimacy than they think, sometimes*” [#3]. There were sentiments that the people who are currently responsible have the capacity to identify research priorities:

“… *And so if you ask me what is our capacity to set priorities we have the capacity. We are able to set priorities on a very objective platform*.” [#10]

However, three respondents felt that the institution was lacking and that the acting office was not strong enough since their legitimacy was undermined by the lack of resources, and weak leadership.

“*My view is that we don’t have a very strong national institution that facilitates priority-setting where everybody is involved and that we’re all buying into that kind of priority-setting that has been set*.” [#9]

### The PS process

Sixteen respondents, including all NGO and the majority of government and university representatives, perceived the consultative, systematic and transparent nature of PS as beneficial to a credible process. However, some respondents (*n* = 12), including some of those who believed in the process, described it as a “*futile exercise*” [#27] in the absence of adequate resources to follow through on commitments made.

#### Stakeholders

All respondents recognised that stakeholder participation was key to successful PS – “*at the end it depends a lot on the mix of the people you have there*” [#7]. Those most referenced represented (external) research funding funders (donors), the Ministry of Health and the Ministry of Finance, research implementers, research institutions, technical planners, medical doctors, and end-users/practitioners (community, partners, district, policy-makers). Additional stakeholders who were less frequently identified included other line ministries, traditional health practitioners and young researchers.

Many stakeholders were identified as necessary to the PS process because of the specific roles that they play within the broader context of health research, healthcare delivery and health systems. Policy-makers and government were seen as funders of research and consumers of research findings, which means that they also have a final say over research priorities in part given their requirement to be accountable to the general public. Government representatives were also seen to help ensure that the priorities set were feasible and the process was appropriate. Researchers not only generate the research and synthesis knowledge, but have to present it in a format that is useful to the policy-makers. The Ministry of Health was identified as the ‘owner’ of the process:

“*So, I guess the Ministry of Health is the top tier, and is the coordinating body for health research in Zambia. So they are ones who basically initiate the whole priority-setting approach, and basically by coordinating and bringing stakeholders on board and saying, ‘Okay, we’ve got this disease area. What are the priorities, and can we establish them so that anybody who wants to do health research kind of buys into this list of priorities that we have?’*” [#22]

The involvement of researchers in particular was seen to be important, which may explain why senior researchers are automatically involved in the PS process. While many were happy to report the presence and involvement of young professionals and students, others felt that the engagement was not fully realised. This was applicable to women as well as younger researchers, even though many felt that there was a gender balance in the PS processes that they had been involved with.

“*In terms of the gender balance and the age, I think we do a fairly good job in Zambia. It’s not that they don’t get a voice*...” [#7]

Seven respondents, mainly from NGOs, consistently identified the lack of practitioners/local experts/NGOs as a weakness in the PS processes that they have been involved with. Many respondents felt that research priorities should be established to help frontline workers and to improve the health of individuals. Several respondents also decried the limited role played by the public:

“… *But I think the missing link, are the beneficiaries of research – basically the communities – there was no community representation. And I think that definitely is a gap because you know, we always take it for granted that the community don’t have much to contribute at the technical level but I think that probably was a gap because we needed the community perspective*…” [#19]

However, some respondents observed that, often times, participation is hampered by the many pressures on peoples’ time and the lack of attention to the time required to complete the process and to ensure that people were available to participate. Respondents also recognised the role played by “*articulate, strong minded*” speakers/advocates in influencing PS; this influence, or bias, is a critical reason for implementing a formal PS process.

The main challenges were centred around communication (“*Internet is… is a constant challenge*” [#2]) and the geography of Zambia (time required to traverse large distances and the number of languages spoken), which make stakeholder consultations difficult and expensive. ICTs were seen as a novel solution to both engagement and data collection.

#### Processes and tools

Most respondents did not refer to the use of a formal PS tool, going as far as to explain that “*there were no tools*” [#15]. Only five respondents referred to specific tools, albeit not necessarily by their formal names, as “*COHRED*”, “*HMIS*”, “*7 processes of priority-setting*”, “*IRS*” tool, and “*CHNRI* [Child Health and Nutrition Research Initiative]”. The tools were not always explained even if the process was followed and they were not pretested. While tools were seen to be useful to support the PS process, it was noted that they need to be applied properly and embedded in policy. Notwithstanding the low use of specific tools, the processes employed were designed to (1) represent the current state of knowledge; (2) solicit broad input to identifying the priorities; and (3) apply criteria for finalising the priorities. Sometimes, not all the steps were followed in all cases:

“… *we never went through any process. All we did was to look at what was the need*.” [#14]

Some respondents recognised the important role that having PS tools played in reducing bias:

“… *There wasn’t any prejudice. There wasn’t any coercion, no room for anyone else to interfere with the framework or the process itself. No one person could have said ‘No, this is the one we’re going to choose’*.” [#3]

#### Criteria

While some of the respondents thought there was no specific criteria, a couple of respondents referred to the criteria within the frameworks they had used in the prioritisation process they were engaged in (discussed above). In addition to these, however, additional considerations were identified, namely the impact of the research and are “*we really going to get value for our money*”, evidence identifying the “*biggest problem*”, “*urgency*”, participants’ experience, gaps in evidence, the national strategic plan, demand, and financial feasibility whereby, if a priority cannot be funded in a given timeframe, it is moved lower on the list:

“*… Yea so you also look at what can you afford? And if you can’t afford it from within your coffers who else can support that activity? Where can you get those kinds of resources? So all those kinds of things play into what you actually decide to adopt as a research activity. So it’s all about what are the issues? How much money do we have? Can we address it right now? If not then kind of move it down. But it’s there on the list*.” [#8]

Mainly due to limited research funds, some of the respondents reported a tendency to prioritise

partners priorities:

“… *Sometimes, because of our limitation of funding, we need… we tend to move towards what partners think are priorities for themselves*…” [#9]

#### Evidence

All respondents saw data and evidence as important for resource allocation processes and for identifying research priorities. The kind of data identified as relevant included the burden of disease, disability-adjusted life years, mortality numbers, causes, available interventions, what should be done, and the kind of research that can be done to better understand and address the issues. However, missing data and lack of an easily accessible centralised source of quality information constrain the use of evidence in decision-making, forcing people to look to other jurisdictions to support evidence-informed decision-making and resulting in incorrect decisions due to challenges with quality control. One-third of respondents (mainly government), identified a need to centralise data for completeness, accuracy and access, and to continue to build capacity in data management and analysis.

“*All this data is held by individuals. So, using ICT platform, can’t we pull all these sources together to a national databank? Because this research, when you do it, it’s not for you. It should benefit the nation... First of all, the data is not stored anywhere for everyone to access. We need an annotated document which shows the type of research, the objectives, the specifics, the findings, summarised… an annotated document.*” [#10]

#### Dissemination of research priorities (publicity)

There are intentions to publicise the national research agenda. However, while five respondents reported that the research priorities were presented in the form of a report to participants in the prioritisation processes, people who were not part of the process did not seem to be able to access the priorities. Ten respondents, mainly university and government representatives, reported that the research priorities are not disseminated very widely, if at all, and often ended up being known only by those that participated.

“*I hear that there was a priority-setting process at the Ministry of Health, however, I’m not exactly sure who was at that table. And after they had set that priority list, it hasn’t been told to us*.” [#15]

#### Availability of mechanisms for appealing (the identified priorities) and enforcing (the process)

No reference was made to specific mechanisms for appealing and making sure that the process adheres to the principles of a fair process.

#### Implementation of set priorities

From a PS perspective, the lack of research funds means that the process can never be seen through to implementation of research findings. More importantly, the majority of respondents (*n* = 24) felt strongly that the identified or perceived domestic research needs are set aside in the face of externally mandated priorities that are attached to research funds. Currently, most health research donors are external to Zambia; hence, respondents called for better domestic funding for health research in order to ensure that national research needs are met. In some cases, researchers have actually turned down research projects despite the presence of funding because they were perceived to be inappropriate within the national context and priorities. Drug trials, in particular, were seen to be problematic. However, regardless of the funding source, there was still a perceived overall disconnect between research funding and research priorities, which may reflect as much on the lack of identified priorities as it does on the lack of resources.

“*…because we’ve been beggars for so many years, we end up getting pushed this direction and that direction by donors*.” [#7]

“*I: to what degree does funding for health research in Zambia reflect the research priorities in Zambia?*

*R: {laughs} I think it doesn’t. No. And if it does then the custodians of it should do a better job of disseminating that information*.” [#19]

There was a sense that it is not uncommon to find that, while health research occurs in Zambia, it does not necessarily align with the identified priorities, but with priorities identified “*elsewhere*” [#7]. There were also concerns raised with regard to the lack of mechanisms for monitoring and evaluation of prioritisation and implementation.

“…*Yeah, there is no monitoring and evaluation mechanism for research that is being implemented all over the country. No.*” [#11]

Hence, respondents proposed a “*basket*” funding approach, where all funders pool the resources which are then used to address identified priorities [#11].

#### Dissensions

Respondents expressed frustrations with regards to the “*lip service*” given to research and policy speeches that are not followed up with implementation [#7].

#### Stakeholder understanding and compliance

While it was difficult to directly assess this parameter, as discussed above, there were sentiments that ‘partners’ tended to not always comply with the research priorities that are identified.

#### Outcome and impact on the PS institution and the Ministry of Health

While there have been PS exercises in Zambia, there has been a lack of designated PS institution. It was hence not possible to assess the impact and outcome of the prioritisation processes on the PS institution. Respondents discussed the difficulties involved in assessing the degree to which PS was successful. This was in part because of a lack of formal monitoring and evaluation programmes, according to eight respondents. One area of success was the convening of a series of research capacity-building workshops.

“*First, we did a proposal development workshop, followed with workshops on data collection, data analysis, and report writing. So, we carried them through the proposal stage, data collection, analysis, and report writing. I think that’s the value of that research agenda. It wasn’t just a document that was lying there*.” [#19]

#### Impact on health policy

While this question was asked in relation to the prioritisation process, some government and university respondents (*n* = 7) discussed the impact of health research evidence on health policy. They decried the fact that, possibly due to poor communication and dissemination of research findings, research evidence does not seem to play an important role in health policy. They identified the need for capacity-building with respect to evaluation metrics of all research projects and programmes. Researcher capacity was required to be able to write and publish peer-reviewed articles and communicate to different audiences, especially with policy-makers, and institutional capacity was required to centralise research findings, make them widely accessible, and translate them into policy and practice.

“*But then once a priority has been set, and research is done, many times the researchers don’t feed back the information to the policy-makers. So, the very essence of that priority-setting is lost because it ends with a research rather than going on to becoming policy and being implemented*.” [#24]

“*We haven’t built the capacity in people, especially the implementers, to understand research and also to do operational research at every level…because some of this doesn’t even need funding. But we haven’t built the capacity of the people who are handling the data.*” [#8]

While the frameworks stipulates that successful PS should result in strengthening of the PS institution and Ministry of Health, respondents identified the need to strengthen the Ministry of Health, particularly to attract greater research funds, and for the government as a whole to provide strong leadership and reduced fragmentation for health research. They proposed that a centralised fund for health research would enhance efficiencies and make it easier for researchers to access and enhance domestic research capacity.

Respondents did not address the following parameters: availability of incentives, efficiency of the process, reduced resource wastage, increased stakeholder (including the public) understanding, improved internal and external accountability, or impact on the health system. Some of these parameters require observation at PS meetings or interviews with the public, which was beyond the scope of the study.

## Discussion

To the best of our knowledge, this is the first paper that provides a detailed evaluation of health research prioritisation in Zambia, using a validated evaluation framework. The qualitative interviews provided some insight into the perceptions of the Zambian stakeholders with regards to PS for health research.

We found that, despite the prioritisation processes that had taken place in Zambia, and some specifically recommending that monitoring and evaluation should be part of the process [7, 8], there did not seem to have been systematic follow-up and evaluation of the implementation of the priorities. This limited opportunities to glean lessons of good practice [17]. Evaluation is critical in facilitating reflexive practice, without which it is difficult for past mistakes not to be repeated [7, 18, 19].

Our study found minimal consistent differences in responses between the respondent categories, which could be due to the fact that all the respondents had participated and had a consistent understanding of the PS process. The few variations in respondents’ responses, e.g. with regards to political will and lack of publicity mechanisms (supported by government and university respondents), human resource availability (supported by donors and university respondents), (university and government respondents) could be explained by the level at which the respondents operate. For example, it is not surprising that Government and university respondents, by virtue of their designated responsibilitites in priority-setting and as government employees who led the prioritisation process and are responsible for publicising the priorities, were more aware of the existence of political will and lack of publicity mechanisms, contrary to the other respondents [17]. However, it is surprising that the donors and university respondents tended to think that the human resources were available while the government and NGO respondents thought otherwise. The explanation given by the latter, namely that they have the “*capacity to attract funding*”, may provide insight into their reasoning.

Among the parameters we were able to evaluate, we found that some would be deemed to have been successful and others less so. Notably, to some degree, the processes described by the respondents seemed to have been participatory and highly consultative; and evidence based. This is critical to increasing the legitimacy and acceptability of the identified priorities. It also reduces the possibilities for dissensions [18, 19]. However, some key stakeholders were not involved in the process. This, especially in terms of the members of the public and representatives from the provinces, could be explained in terms of feasibility and level of expertise. While there should be efforts to ensure that the views of these stakeholders are represented, it may not be possible to directly engage all stakeholders. Publicising the priorities with their rationales would contribute to bridging this gap [19–21]. However, considering that Zambia is decentralised, national priorities ought to include research priorities that are identified within the respective provinces, as proposed by Kapiriri et al. [18, 21]. Participation, if well designed, could also deal with the problem of the ‘partners’ lack of aligning their funding with the identified priorities.

While there is a strong political will as evidenced by the support for setting up a research authority, at the time of the study, the Ministry of Health was still in the process of operationalising the National Health Research Authority of Zambia. The lack of a fully functional Authority made it impossible to evaluate the parameters related to the institute. Since the authority has been operational since 2017, it should ensure that clear, explicit and fair prioritisation processes and explicit criteria are instituted, including mechanisms for publicising the identified priorities, appeals, revisions, implementation and evaluation, in order to strengthen PS. The reference manual developed by the authors could provide guidance [21]. From the start, it is critical that the institute has the capacity and resources necessary to not only identify priorities, but to implement identified priorities (for example, through open calls, where they fund priority areas) and evaluate the impact of their processes as well as ensuring that the research findings are available to policy-makers [6]. As discussed by the respondents, and consistent with the literature, in addition to funding the research, research funders should also support the researchers and oblige them to translate and communicate their research findings in an accessible manner to the various relevant stakeholders [7]. This will facilitate use of research and motivate further buy-in from researchers and policy-makers as well. Ideally, research priorities should be informed by pressing issues in policy-making and practice and the research findings should subsequently inform them.

We recognise that the above activities cannot replace the need for research funding. Our findings that a lack of designated funding for health research introduces actors whose priorities may not align with national priorities are not unique to Zambia, or health research [8, 22]; hence, there is a need for the government and partners to ensure that there are funds to contribute to the strengthening and enabling of the new research authority to carry out its duties as well as funding for the identified health research priorities. Actual allocation of funds to the identified priorities will close the loop from PS to implementation and impact [7, 22].

## Limitations

The inability to evaluate all the parameters in the framework can, in part, be explained by the absence of a fully functioning PS organisation/authority, as discussed above, and also due to the identified means of verification. While in the validation process these means of verification were thought to be feasible, the approach used in the study was not able to yield this information. The means of verification for most of these parameters necessitated real time data collection such as observations of PS meetings and exit interviews, which were not feasible at the time of the study. This calls for integrating evaluation in the prioritisation and monitoring process to ensure that the required information is collected [18, 21, 22]. A follow-up study to assess progress made in addressing the identified challenges in systematic PS for health research is recommended.

Furthermore, while we endeavoured to enlist responses from sub-national levels, most of the respondents were from the national level, hence the voices from the provinces and districts are under-represented. Second, even at the national level, the sample size and nature of the interviews means that we cannot claim that our findings are generalisable. However, since we endeavoured to include almost all participants in the prior prioritisation processes, the findings are credible since they represent their experiences.

## Conclusions

While Zambia has undertaken several health research prioritisation exercises, no systematic evaluation of the processes had been performed; the present study filled this gap, using a validated framework. The framework for evaluating health research PS identified the bottlenecks in health research in Zambia as well as opportunities to not only redress these, but also formalise a standardised national PS process applied at all levels for all health-related decision-making. A key finding is that the foundation laid through enactment of the Health Research Act is extremely timely. The Act provides a legal framework for formalised processes, streamlined resources and funding allocation, centralised reporting and data storage, and access, and translation of research findings into policy and practice. However, for this to be realised, the funding challenge must be resolved, as it has implications not only for the PS process, but also for purposes of legitimacy and ensuring that domestic research needs are met. Ultimately, there is a clearly articulated need for understanding PS processes, benefits of formal PS processes and the importance of health research in Zambia.

This is the first occasion in which the evaluation framework has been applied to evaluate a case of PS. The study highlights the need for integrating evaluation in the planning phase of the prioritisation process. While the similarities between Zambia and other low-income countries make the results relevant to these countries, there is a need for additional case studies of application of the framework to identify any unique experiences.

## Abbreviations

NGOs: non-governmental organisations
PS: priority-setting
